# Music and Visual Art Training Increase Auditory-Evoked Theta Oscillations in Older Adults

**DOI:** 10.3390/brainsci12101300

**Published:** 2022-09-27

**Authors:** Jennifer A. Bugos, Gavin M. Bidelman, Sylvain Moreno, Dawei Shen, Jing Lu, Claude Alain

**Affiliations:** 1 School of Music, University of South Florida, Tampa, FL 33620, USA; 2 Department of Speech, Language, and Hearing Sciences, Indiana University, Bloomington, IN 47408, USA; 3 School of Interactive Arts and Technology, Simon Fraser University, Burnaby, BC V3T OA3, Canada; 4 Circle Innovation, Burnaby, BC V3T OA3, Canada; 5 Rotman Research Institute, Toronto, ON M6A 2E1, Canada; 6 MOE Key Lab for Neuroinformation, School of Life Science and Technology, University of Electronic and Science Technology of China, Chengdu 611731, China; 7 Department of Psychology, University of Toronto, Toronto, ON M5S 3G3, Canada

**Keywords:** time-frequency, neural oscillations, older adult, music, visual art, theta

## Abstract

Music training was shown to induce changes in auditory processing in older adults. However, most findings stem from correlational studies and fewer examine long-term sustainable benefits. Moreover, research shows small and variable changes in auditory event-related potential (ERP) amplitudes and/or latencies in older adults. Conventional time domain analysis methods, however, are susceptible to latency jitter in evoked responses and may miss important information of brain processing. Here, we used time-frequency analyses to examine training-related changes in auditory-evoked oscillatory activity in healthy older adults (*N* = 50) assigned to a music training (*n* = 16), visual art training (*n* = 17), or a no-treatment control (*n* = 17) group. All three groups were presented with oddball auditory paradigms with synthesized piano tones or vowels during the acquisition of high-density EEG. Neurophysiological measures were collected at three-time points: pre-training, post-training, and at a three-month follow-up. Training programs were administered for 12-weeks. Increased theta power was found pre and post- training for the music (*p* = 0.010) and visual art group (*p* = 0.010) as compared to controls (*p* = 0.776) and maintained at the three-month follow-up. Results showed training-related plasticity on auditory processing in aging adults. Neuroplastic changes were maintained three months post-training, suggesting music and visual art programs yield lasting benefits that might facilitate encoding, retention, and memory retrieval.

## 1. Introduction

Music training is a temporally complex activity that contributes to auditory processing and neuroplasticity [1]. Practicing a musical instrument builds a sensory vocabulary of internal representations to generate predictions about upcoming sensory input [2]. For instance, temporal elements are necessary to predict the following rhythmic pattern for the listener, and prediction is critical to the timing and articulation of sounds that emerge during instrumental performance [3]. Given the reliance upon sensory predictions in some learning domains, it is increasingly common for researchers to use neurophysiological measurements that capture time-frequency information [4]. However, most research in music training focuses upon conventional measures of event-related potentials (ERPs) that are susceptible to latency jitter in evoked responses. These measures may not capture neural activity during temporally complex tasks. Neural oscillations provide a complementary measure of auditory processing and neural plasticity not available in conventional (e.g., ERP) techniques. This aim of this study was to examine the effects of a novel music and visual art training programs on auditory evoked and induced oscillatory activity in aging adults.

### 1.1. Predictive Coding Theory and Auditory Segmentation

Predictive coding theory suggests that generated predictions from actual sensory input guide the brain’s internal states compared with previous information gained through experiences [5]. The brain may respond to sound objects against a priori sensory predictions in various elements of music, i.e., rhythm and meter [6]. Research also suggested that theta oscillations reflect an active auditory segmentation mechanism that may complement auditory entrainment [7]. According to this hypothesis, incoming acoustic information within a temporal window of 150 to 300 ms would be grouped together. The process of chunking incoming streams of sound events is thought to facilitate the prediction and identification of sound objects. If this is the case, music training may strengthen the auditory system’s ability to perceptually organize sound events. In addition, changes in oscillatory power are assumed to reflect the size of the neuronal assembly involved in stimulus processing and temporal synchronization [8]. We hypothesized that music training might increase auditory segmentation resulting in increased theta activity when compared to visual art training or no treatment controls.

Several EEG studies applying time-frequency analysis have revealed differences in oscillatory neural activity in listeners with music experience [9,10]. Cross-sectional studies comparing musicians and non-musicians showed lower spectral coherence in overall neural oscillations over temporal, central, and parietal scalp locations while completing a pattern recognition and spatial working memory task [9]. Similarly, musicians with absolute or relative pitch show decreased cortical activity over temporal and parietal regions compared to non-musicians [10]. Moreover, several intervention studies examining music training in children [11,12,13,14] and young adults [15] have observed changes in induced brain responses in auditory and motor regions [16]. Relevant to this study, several cross-sectional reports have shown enhancements in both evoked and induced neural oscillations related to improved perceptual-cognitive function, including those related to speech perception [17,18,19]. For instance, research showed differences in gamma, alpha, and beta activity in young adults with music training as compared to controls. Musicians demonstrated stronger auditory object representations for speech signals and were able to match sounds to internalized memory templates [17]. Shahin and colleagues [18] found gamma band activity for professional adult musicians with larger bursts associated with the instrument of specialization. Research examining gamma activity in professional musicians and young children also found enhanced gamma activity in adult musicians [19]. In contrast to professional musicians, no significant evoked gamma responses were found for children (4–5 years) who were beginning music instruction. However, to date most of these studies were cross-sectional or were restricted to children and young adults. Whether or not such neuroplasticity is causally related to music training and exists in older adults has yet to be determined.

### 1.2. Neural Oscillations in Aging

Aging is associated with impaired hippocampal-dependent learning characterized by reduced theta oscillations [20,21]. Theta oscillations rely upon the supply and reuptake of cholinergic input and are critical for novel learning and memory processes, e.g., encoding/recognition, working memory, and episodic memory [22,23]. For instance, memory recollection tasks in older adults show reductions in theta and beta oscillatory power during stimulus processing, and post-stimulus theta activity positively correlates with episodic memory performance [24,25]. Evidence also suggests that theta oscillations are sensitive to the temporal order of stimulus events and are less relevant to storage function in learning [26]. These data have broad implications for theta oscillations as a potential metric to index learning and memory and may provide a means to examine how older adults respond to training programs with the aim of improving or offsetting age-related declines in cognitive function.

Moreover, different frequency rhythms of the EEG are not homogenous but might be related to different perceptual and cognitive functions. This leads to the intriguing possibility that music training may be associated with changes in lower vs. higher oscillatory bands depending on the nature of the stimuli and task demands [27]. Indeed, differences between cortical responses underlying pitch and duration processing in young adult musicians and non-musicians indicate experience-dependent changes in neural activity [28,29]. Oscillatory brain activity and long-range intra-hemispheric coherence in theta band was significantly higher during a verbal memory task for musicians than non-musicians, despite limited group differences in intra- and inter-hemispheric coherence during encoding [30]. Increased theta power over the right frontoparietal scalp has also been observed in adults learning short piano sequences [31]. These findings suggest that theta power may be associated with reconciling a prediction with an unfamiliar sound response. Collectively, these studies suggest associations between neural oscillations in the theta band and musical expertise.

Low-frequency theta oscillations are also associated with enhanced executive functions in sustained attention [32], working memory [33], and memory encoding [34]. Research examining age-related differences found that theta activity was associated with attention refocusing on tasks requiring consolidation [35]. Researchers found differences in theta recruitment with increased load on auditory working memory tasks (e.g., N-Back), with increased mid-frontal theta power for young adults, and decreased theta power in older adults [36].

Oscillations in higher EEG bands, namely the beta frequency range, have been linked to pitch processing and are similarly enhanced in musicians [27]. Beta oscillations have also been associated with rhythmic regularity and timing of movements in musicians [37,38]. For instance, beta signal power decreases have been found before and during movements, while increases in the strength of beta have been found after movements [39]. Research examining beta-band oscillations during a passive listening task in older adults found modulated beta oscillations in the auditory cortices similar to younger adults after 15 piano lessons [40]. Beta power was linked to prediction error and prediction updating after the onset of an unexpected sensory event [2,41]. In addition, the results of an auditory oddball task showed a U-shaped beta power modulation on the left auditory cortex and delta-beta coupling on the right auditory cortex [29]. Researchers examined the effects of 15 individualized piano lessons in seven healthy adults on beta and gamma oscillations found increased gamma synchrony post-training and a shift of dipole source locations toward the anterior direction compared to participants who did not receive piano training [42]. Yet, no studies, to our knowledge, have evaluated the effects of general music training on neural oscillations in aging adults compared to an active control condition, visual art training, or no-treatment group.

To this end, the purpose of this study was to examine evoked oscillatory neural activity in older adults who were assigned to either music training, visual art training, or a no-treatment control group. We sought to extend previous findings from Alain and colleagues [43] by applying time-frequency analyses to assess the effect of training on oscillatory power indexing auditory deviance processing of speech (vowel oddball) and nonspeech (pitch oddball) sounds. Since pitch changes and sensorimotor representations in music have been associated with beta and theta oscillations, we hypothesized an increase in theta and beta power post-training as compared to visual art (active control) or a passive (no intervention) control group [28,30,40]. For those enrolled in visual art training, we hypothesized an increase in gamma power would emerge post-training, given previous associations between gamma and visual orientation [44].

## 2. Materials and Methods

### 2.1. Participants

We examined oscillatory neural activity in older adults over age 60 (*N* = 50, Table 1). Adults consisted of those who were randomly assigned to a music training (*n* = 16) or visual art training (*n* = 17) group and a group of adults who were separately recruited for a no treatment control (*n* = 17) condition [43]. New measures reported herein extend the findings of Alain and colleagues [43] by examining auditory deviance processing for speech and non-speech sounds. Participants had limited prior music or art training and were previously screened for amusia and auditory deficits with the Musical Ear Test [45]. Demographic characteristics across groups (Table 1) showed no differences in age, years of education, or estimation of intelligence as measured by the Wechsler Abbreviated Scale of Intelligence-Second Edition [43,46]. Written informed consent was obtained from all participants in conjunction with the policies established by the Baycrest Research Ethics Committee.

All participants had limited music and visual art training: They had not engaged in any visual art or musical training or activity within the past five years and had no more than six years of formal visual arts or musical training over their lifespan. The music group had an average of 1.2 years (*SD* 2.0) of private music lessons and 0.7 years (*SD* 1.2) of private art lessons, and the art group had an average of 1.3 years (SD 1.8) of private music lessons and 0.2 years (*SD* 0.7) of private art lessons. The control group had 0.7 years (*SD* 1.1) of private music lessons and 0.3 years (*SD* 0.9) of private art lessons.

### 2.2. Procedure

Participants completed a battery of measures, pre-, and post-training, as described in Alain and colleagues [43]. The battery of cognitive measures included the Wechsler Abbreviated Scale of Intelligence-Second Edition, Forward and Backward Word Span, Stroop Test, Computerized Peabody Picture Vocabulary Test, and the Digit symbol (subtest of the WAIS-R). The findings from these are presented in Alain et al. [43]. During the pre-, post-, and follow-up training sessions, participants were presented with a visual Go-NoGo paradigm and two auditory oddball paradigms. Here, we report neural oscillations while participants were presented with two auditory oddball paradigms (pitch and speech).

**Figure 1:**
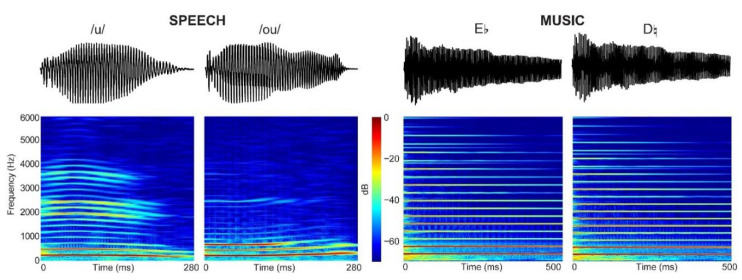
Spectrograms of the auditory stimuli for speech and music oddball paradigms.

Both oddball paradigms included 510 standards and 90 deviant sounds presented in a pseudorandomized order, with the constraint that two deviants could not be presented consecutively. The standard and deviant stimuli were matched in acoustic features, including amplitude and duration. The stimulus onset asynchrony was 1250 ms. The binaural stimuli (see Figure 1) were presented at 80 dB sound pressure level via ER-3 insert earphones (Etymotic Research). In the pitch auditory oddball paradigm, participants were presented with two 500-ms synthesized piano tones that differed only on their fundamental frequency (F0) (standard: Eb4, F0 = 314 Hz; deviant: D4, F0 = 294 Hz). In the speech oddball paradigm, participants were presented with two natural vowels recorded from a native female French speaker. The standard (/u/) and deviant (/ou/) vowels were edited to have the same duration (280 ms). They also had comparable mean voice F0, and amplitude. The two speech token differed primarily on their second formant (standard: /u/∼1850 Hz; deviant: /ou/∼750 Hz), which yielded two distinct vowel timbres.

### 2.3. Description of Training Programs

Training classes met three times per week for one-hour (total of 36 h). A qualified visual art and music instructor taught courses at the Royal Conservatory of Music. Music training included a general music curriculum with body percussion, voice, and non-pitched percussion instruments. Musical concepts of melody and harmony were demonstrated through singing activities (e.g., canons, rounds). The visual art curriculum included basic drawing and painting techniques, the creation of original works, and the analysis of masterworks. Participants received all materials for their respective courses, and no outside practice was required.

### 2.4. ERP Data Collection

A BioSemi Active Two acquisition system (BioSemi, V.O.F., Amsterdam, The Netherlands) was utilized to record electrophysiological (EEG) data. We used a BioSemi electrode cap based on the 10/20 system [47] with a common mode sense active electrode and a passive ground electrode on the right leg. To cover the entire scalp evenly and monitor eye movements, we applied 10 additional electrodes below the hairline (both mastoid, pre-auricular points, outer canthus for each eye, inferior orbit of each eye, and two facial electrodes). Data from 76 electrodes were digitized continuously at 512 Hz with a band-pass of DC-100 Hz. Analyses were conducted offline using Brain Electrical Source Analysis Software (BESA, version 7.0: MEGIS GmbH, Grafelfing, Germany).

The EEG data were visually inspected to identify segments contaminated by defective electrodes. Noisy electrodes were interpolated using data from the surrounding electrodes, and no more than eight electrodes were interpolated per participant. The EEG was then re-referenced to the average of all electrodes and digitally filtered using a high-pass filter with a cut-off frequency of 1.0 Hz (forward, 6 dB/octave). For each participant, a set of ocular movements was identified from the continuous EEG recording and then used to generate spatial components to best account for eye movement artifacts. The spatial topographies were then subtracted from the continuous EEG to correct for lateral and vertical eye movements as well as for eye blinks. The data were parsed into 900 ms epochs time-locked to stimulus onset, including 300 ms of pre-stimulus activity. Epochs with artifact deflections exceeding ± 60 µV were marked and excluded from further analysis.

### 2.5. EEG Time-Frequency Preprocessing

The conversion of continuous EEG data into the time-frequency domain was performed with BESA Research 7.0. We used a complex demodulation method with 2 Hz wide frequency bins and 25 ms time resolution from −300 to 900 ms (with zero-padding of 2000 ms) in the range of 4 and 50 Hz for decomposing the single-trial EEG data into a time-frequency representation of temporal spectral evolution (TSE, an equivalent measure of event-related (de)-synchronization) to quantify the change in oscillatory power over time). The mean oscillatory activity between −250 to −50 ms was used for baseline correction. These parameters were chosen to ensure sufficient temporal resolution to examine sensory evoked responses and mismatch negativity while maintaining good frequency resolution.

### 2.6. Statistical Analyses

The effects of stimulus type (i.e., deviant vs. standard) on auditory evoked potentials and the corresponding oscillatory brain activity were subjected to cluster-based permutation testing using BESA Statistics (Statistics 2.0, MEGIS GmbH, Gräfelfing, Germany). This data-driven analysis does not require a priory decision regarding when and where over the scalp the deviance-related brain activity is best expressed in healthy older adults. BESA Statistics 2.1 software automatically identifies clusters of electrodes in space, time, and frequency that significantly differ between deviant and standard stimuli. A Monte-Carlo resampling technique [48] is then used to identify significant clusters (i.e., above chance level) by random data permutation. Importantly, BESA Statistics 2.1 corrects for multiple comparisons across time, frequency, and electrodes. An alpha of 0.05 was used for cluster building. The number of permutations was set at N = 3000.

The clustered-based statistic was first performed on auditory evoked potentials from 0 to 400 ms post-stimulus using 61 scalp electrodes. We excluded electrodes below the hair line (i.e., F9, F10, FT9, FT10, TP9, TP10, P9, P10, CB1, CB2, Iz), electrodes lateral and below the eyes (i.e., LO1, LO2, IO1, IO2), which tend to be noisier than scalp electrodes and are more sensitive to muscle artifacts. Separate cluster-based statistics were performance for the auditory evoked responses from the music and speech oddball paradigms. These analyses comprised all participants from the first EEG session and aimed to identify the time course of the mismatch negative (MMN) potential.

The effect of training on oscillatory-evoked activity was examined using a priori planned contrasts within the repeated measured ANOVA with the Visual Art group as our reference category and session as the within-subject variable. Our primary hypothesis was that the effect of treatment will differ between the experimental group (i.e., Music) and the active control group (i.e., Visual Art). The secondary hypothesis was that the active control group would differ from the passive control group.

## 3. Results

**Figure 2:**
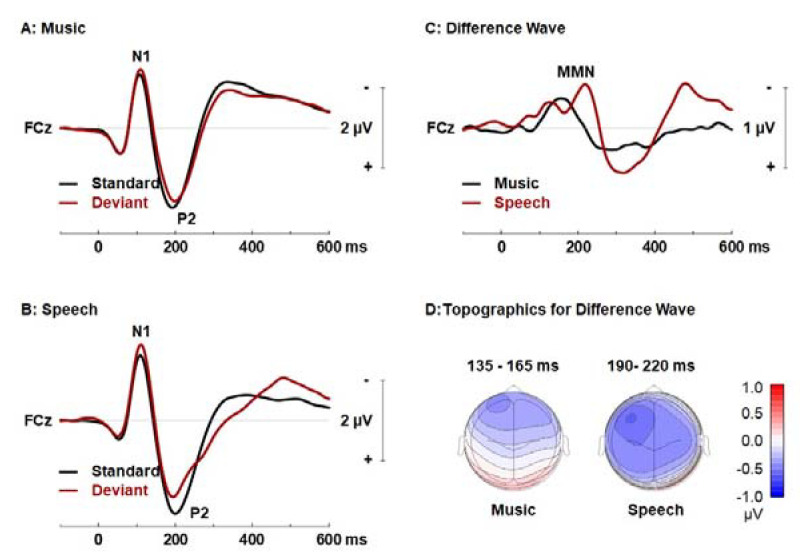
Pooled group mean auditory evoked responses elicited by the standard and deviant sounds in the music (**A**) and speech (**B**) oddball paradigms at pre-test. The difference waves used to isolate the mismatch negativity (MMN) are shown on the panel (**C**,**D**) Isocontour maps showing the MMN mean amplitude distribution for a 30 ms interval. FCz = frontocentral electrode.

Figure 2 shows pooled group mean auditory evoked responses elicited by the standard and deviant sounds, and the corresponding difference wave isolating the MMN in the music and speech oddball paradigm before music and visual art intervention program. Alain et colleagues [43] previously reported MMN responses peaking earlier for music as compared to speech stimuli. Training groups were previously reported to have an earlier peak latency as compared to controls (i.e., training effect). Furthermore, the amplitude of the MMN for speech was larger than that of the music oddball task. Here, we expand upon these findings with cluster-based statistics.

Clustered-based statistic revealed a significant MMN in the music paradigms, which peaked at 145 ms over frontocentral scalp areas (Table 2). The MMN response was significant between 90 and 265 ms following deviant onset over frontal and frontocentral sites. It was followed by a small positive deflection peaking at 370 ms over the right central scalp area. There was also a significant MMN in the speech oddball paradigm. The clustered-based statistic revealed a significant MMN response between 50 and 330 ms post-stimulus (Table 2). The MMN peaked at 213 ms after deviant onset over the left frontal scalp area and was followed by a positive deflection peak at 360 ms post-deviant onset over the left temporal-parietal cortex. In the next section, we examine oscillatory-evoked activity that best correlate with the MMN response recorded in both music and speech oddball paradigms.

### 3.1. Effects of Training on Oscillatory Activity Indexing Auditory Processing

**Figure 3:**
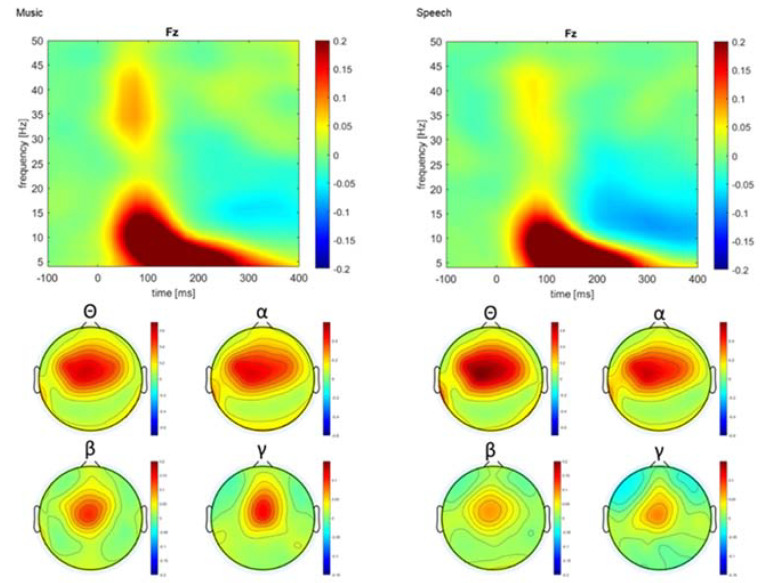
Pooled group mean power temporal spectral evolution (TSE) elicited by standard stimuli at the midline frontal electrode (Fz), and the corresponding scalp distribution for theta (θ), alpha (α), beta (β), and gamma (γ) power at 150 ms, 100 ms, 100 ms, and 75 ms post-stimulus, respectively.

**Figure 4:**
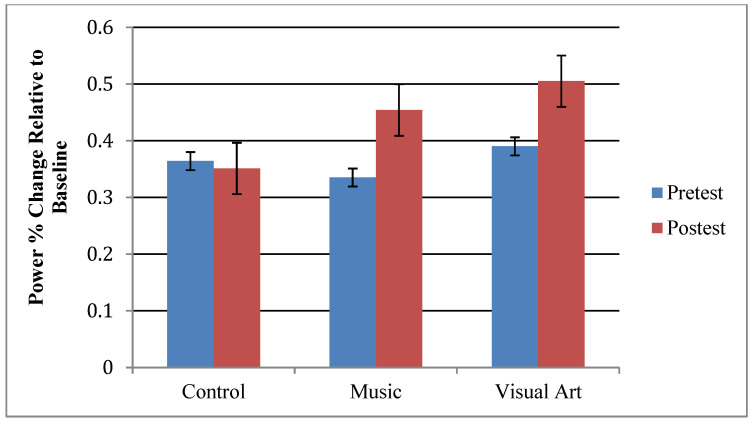
Group mean theta power elicited by the standard stimuli during pre-training (Session 1) and post-training (Session 2).

In both paradigms, standard stimuli were associated with an increase in theta, alpha, beta, and gamma band power between 75 and 225 ms post-stimulus, which was typically largest over the mid frontal area (Figure 3). The effects of training on the theta power were examined for the 100–175 ms interval at frontal sites (F1, Fz, F2) using mixed model repeated measure ANOVA including paradigm (music, speech) and session as within-subject factor, and group as between factor. The ANOVA yielded a main effect of session, *F*(1,47) = 8.67, *p* = 0.005, *ηp*^2^ = 0.156, with greater theta in the post- than in the pre-training session. The planned contrast between experimental group and active control group did not yield a significant group x session interaction, *F*(1,47) = 1.55, *p* = 0.219, *ηp*^2^ = 0.032. However, the contrast between active and passive control group yielded a significant group x session interaction, *F*(1,47) = 5.96, *p* = 0.018, *ηp*^2^ = 0.113. Pairwise comparisons revealed increased theta power at post-test in those who received music training (*p* = 0.010) or visual art training (*p* = 0.010). There was no significant difference between pre-test and post-test in older adults from the control group (*p* = 0.776). The main effect of the paradigm was not significant (*F* < 1) (Figure 4).

The analysis of alpha power measured at mid-frontal areas (F1, Fz, F2) revealed a trend suggesting greater power at post- than at the pre-training session, *F*(1,47) = 4.04, *p* = 0.050, *ηp*^2^ = 0.079. The alpha power tended to be larger during the post-training session than the pre-training session in the experimental group compare to the active control group (group x session interaction: *F(*1,47) = 3.32, *p* = 0.075, *ηp*^2^ = 0.066). Similarly, alpha power tended to be larger at post-training in the active control group than in the passive control group (group x session interaction: *F*(1,47) = 3.15, *p* = 0.083, *ηp*^2^ = 0.063). Lastly, music stimuli generated greater alpha power than speech sounds, *F*(1,47) = 14.38, *p* < 0.001, *ηp*^2^ = 0.234). For beta power measured at frontal sites (F1, Fz, F2), the main effect of session was not significant (*F* < 1), nor was the interaction between session and group (*F* < 1 in both cases). However, music stimuli generated greater beta power than speech sounds, *F*(1,47) = 14.11, *p* < 0.001, *ηp*^2^ = 0.231. Similarly, for the gamma power measured at frontal sites (F1, Fz, F2), the main effect of session was not significant nor was the interaction between session and group. Music stimuli generated greater gamma responses than speech sounds, *F*(1,47) = 13.89, *p* < 0.001, *ηp*^2^ = 0.228).

Overall, our results showed increased theta power associated with processing standard music and speech stimuli during the post-training session for those who received music and visual art training as compared to controls. While alpha increased for those in the music group as compared to the visual art group, both training groups demonstrated increased alpha power when compared to controls. Lastly, music stimuli generated greater alpha, beta, and gamma responses than speech sounds.

### 3.2. Brain Oscillations Indexing Automatic Deviance Detection: Pre-Training

**Figure 5:**
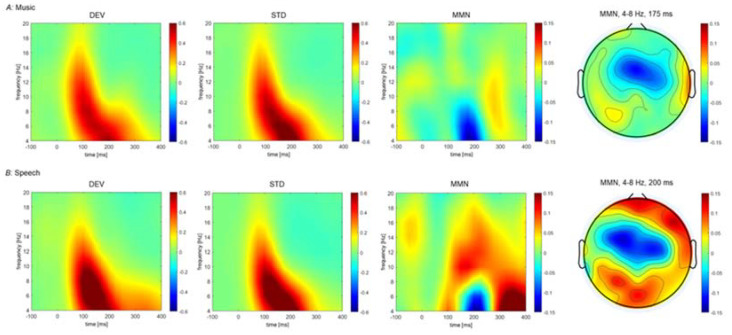
Grand average power temporal spectral evolution (TSE) elicited by standard and deviant stimuli, and the corresponding difference during the music oddball paradigm. (**A**) Time-frequency plots for deviant (DEV), standard (STD) stimuli, and the corresponding different wave used to isolate the mismatch negativity (MMN). (**B**) Time-frequency plot for the vowel stimuli.

We first compared oscillatory brain activity elicited by the standard and deviant sounds from the music and speech oddball paradigms among all participants (Figure 5). For the music paradigm, the processing of the standard and deviant stimuli was associated with oscillatory-evoked activity in the theta, alpha, and beta band between 80 and 220 ms post-stimulus. The contrast between oscillatory activity elicited by deviant and standard stimuli revealed lower theta power (4–8 Hz) for the deviant than standard stimuli over the frontocentral scalp areas, which was accompanied by enhanced theta power over the right temporal cortex (Table 3). There was no difference between standard and deviant stimuli for alpha power. Deviant stimuli yielded lower beta power than standard stimuli over the frontal scalp areas (Table 3). There was no difference in gamma power elicited by deviant and standard stimuli.

For the speech oddball paradigm, processing of standard and deviant speech sounds was associated with enhanced evoked oscillatory activity in theta, alpha, and beta band between 60 and 300 ms post-stimulus. The clustered-based permutation statistic revealed greater theta power for deviant than standard stimuli over right fronto-temporal-parietal cortex and over the left parietal cortex (Table 3). Occasional deviant speech sounds also generated greater alpha power than frequent speech stimuli. There was no difference in beta power between deviant and standard stimuli. Lastly, deviant speech sounds generated enhanced gamma activity relative to standard sounds over the frontocentral scalp areas (Table 3).

### 3.3. Music Oddball Paradigm: Effects of Training on Deviance-Related Oscillatory Activity

The contrast between oscillatory activity elicited by the deviant and standard stimuli revealed changes in theta, beta, and gamma bands. The effect of training on the mean theta power was examined for the 150–225 ms interval at frontocentral (FC1, FCz, FC2) and parietal-occipital sites (PO3, POz, PO4). For the frontocentral region of interest (ROI), the ANOVA on the mean power yielded a main effect of session, *F*(1,47) = 15.46, *p* < 0.001, *ηp*^2^ = 0.248, and a significant session x stimulus type interaction, *F*(1,47) = 18.54, *p* < 0.001, *ηp*^2^ = 0.283. The session x stimulus type x group interaction was not significant, *F*(2,47) = 2.35, *p* = 0.106, *ηp*^2^ = 0.091. For the right temporal ROI, the ANOVA yield a main effect of stimulus type, *F*(1,47) = 21.23, *p* < 0.001, *ηp*^2^ = 0.311. No other main effect or interactions were significant.

The effects of training on deviance-related changes in beta power was examined for the 175–250 ms interval over the anterior frontal scalp areas (AF3, AFz, AF4). The main effect of stimulus type was significant, *F*(1,47) = 6.12, *p* = 0.017, *ηp*^2^ = 0.115. The main effect of session was not significant, *F*(1,47) = 2.38, *p* = 0.130, *ηp*^2^ = 0.057, nor was the session x stimulus x group interaction (F < 1). The session x stimulus type interaction trended toward significance, *F*(1,47) = 3.77, *p* = 0.058, *ηp*^2^ = 0.074. Thus, changes in oscillatory activity do not appear to be affected by music or visual art training.

### 3.4. Speech Oddball Paradigm: Effects of Training on Deviance-Related Oscillatory Activity

The contrast between oscillatory activity elicited by the deviant and standard speech sounds revealed changes in theta, alpha, and gamma band. The effect of training on the mean theta power was examined for the 150–225 ms interval at frontocentral (FC1, FCz, FC2) and parietal-occipital sites (PO3, POz, PO4). For the frontocentral ROI, the ANOVA yielded a main effect of stimulus type, *F*(1,47) = 6.13, *p* = 0.017, *ηp*^2^ = 0.115. No other main effect or interactions were significant. For the parietal-occipital ROI, the ANOVA also yielded a main effect of stimulus type, *F*(1,47) = 5.09, *p* = 0.029, *ηp*^2^ = 0.098. The main effect of session was not significant, *F*(1,47) = 1.31, *p* = 0.258, *ηp*^2^ = 0.027. There was a significant session x stimulus type interaction, *F*(1,47) = 9.05, *p* = 0.004, *ηp*^2^ = 0.161. The session x stimulus type x group was not significant (*F* < 1).

The effects of training on deviance-related changes in alpha power were examined for the 150–225 ms interval over parietal-occipital (PO3, POz, PO4) area. There was a main effect of stimulus type, *F*(1,47) = 19.24, *p* < 0.001, *ηp*^2^ = 0.290. No other main effects or interactions were significant. The effects of training on deviance-related changes in gamma band activity were assessed for the 100–175 ms interval over the left parietal area (P3, P5, P7). The main effect of stimulus type was significant, *F*(1,47) = 5.07, *p* = 0.029, *ηp*^2^ = 0.097. No other main effect or interactions were significant. These data showed differences for stimulus type; however, music and visual art training did not significantly modulate theta, alpha, or gamma power.

### 3.5. Follow-Up

**Figure 6:**
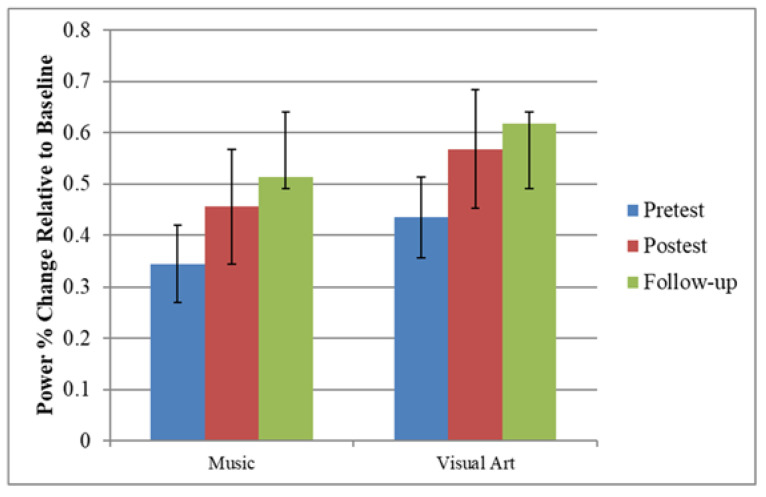

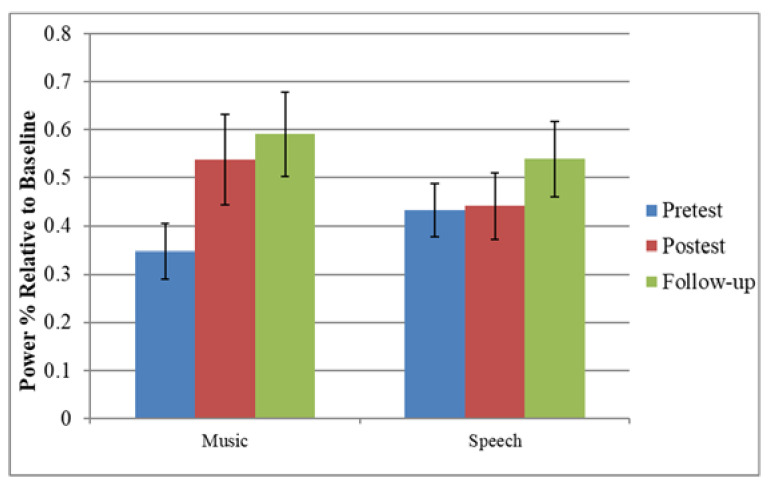
(Top panel) Theta power by group as a function of session; and (Bottom panel) Theta power as a function of stimuli presented by session.

A subset of participants from the music (*n* = 14) and visual art (*n* = 13) training groups took part in a three-month, post-training follow-uptime-point. Here, we tested whether the enhanced theta power following music and visual art training was long-lasting. In Figure 6, the top panel shows the group means theta power elicited by the standard stimuli as a function of the session. The analysis revealed a main effect of session, *F*(2,50) = 9.195, *p* < 0.001, *ηp*^2^ = 0.269). The pairwise comparison revealed enhanced theta power at post-test and follow-up relative to pre-test (*p* < 0.01 in both cases). There was no significant difference between post-test and follow-up (*p* = 0.198). The interaction between group and session was not significant (*F* < 1). The analysis also revealed a significant interaction between paradigm and session, *F*(2,50) = 9.70, *p* < 0.001, *ηp*^2^ = 0.280. For the music stimuli, theta power increased significantly between the pre-training and post-training, and then showed little changes in power. For the speech sounds, theta power increased slightly from pre-training to post-training, but then showed a more pronounced increase between post-training and follow-up session (Figure 5 bottom panel).

## 4. Discussion

The present study aimed to examine the causal effects of music and visual art training on neural oscillations in older adults. Critically, groups did not differ in automatic change detection at pre-test, as evidenced by their similar MMN and time-frequency responses before training.

To examine intervention effects, we analyzed the change between pre- and post-training oscillatory power. Time-frequency analysis revealed differences in theta power elicited by piano tones for those who completed music or visual art training compared to controls. These increases were found in early sensory processing around 100–175 ms at frontal electrodes. These results are consistent with research in children [11,13], and extend our understanding of neural plasticity after music or art training to the aging brain.

Aging is often associated with reductions in theta recruitment when compared to younger adults [49]. Theta oscillations in mid-frontal regions have been associated with cognitive control and “chunking” of novel perceptual auditory information [4,50]. Due to the novelty of the harmonic stimuli (i.e., piano tones), it is possible that participants allocated attention to the unfamiliar piano tones. Prior work suggests that theta indexes goal-directed responses. In the present study, changes in theta power may reflect a release from habituation through a process of phase resetting, which may capture attention [22] and engage cognitive control mechanisms [50].

In addition to theta oscillations, we found differences in alpha power for those who received music or visual art training as compared to controls. Most notably alpha was more prominent for the music condition as compared to the speech condition. Alpha oscillations have been associated with selective suppression of task-irrelevant information [51]. Studies comparing different modalities (e.g., visual, auditory, audio-visual oddballs) found that young adults were able to ignore irrelevant visual information contributing to auditory attention, which resulted in continuous alpha phase oscillations [52]. Participants in the present study watched a silent video while passively listening (no response required) to piano tones or speech sounds. While theta and alpha oscillations are associated with attention allocation with respect to sensory stimuli, oscillatory activity in these frequencies may include different mechanisms that may be influenced by learning. One possibility is that alpha may index sensory processing of distracting information, and oscillatory activity in the theta band may contribute to other areas of brain function through resetting attention [52,53].

Older adults in the current study may have allocated additional cognitive resources to process novel stimuli in the pitch condition as compared to the speech condition. To recruit additional cognitive resources, adults needed to suppress the processing of task-irrelevant stimuli (e.g., a silent movie) while passively completely the auditory oddball tasks. This explanation aligns with other research accounts, which suggest the need to ignore visual surroundings in a continuous manner to enhance the processing of auditory sequences [52].

Music and visual art training may have enhanced or contributed to the deployment of cognitive resources that allowed for higher neural efficiency post-training. Sensory processing, common to both general music and visual art training, could build additional scaffolding necessary to assist with attentional processing [54]. In addition, increases in neural efficiency were maintained at the follow-up time point, demonstrating that these interventions may contribute to enhance cognitive and sensory processing. Data are consistent with other studies in adults that suggest neural oscillatory activity relates to the strength of memories formed in learning [55,56]. Furthermore, our findings related to increases in theta are consistent with other studies examining sequence processing in music [30,31] and those related to focused attention in meditation [57].

Our data showed enhancement of theta during the pitch condition but suppression of theta for the speech condition. This finding may be related to acoustic differences between the piano and the speech sounds, including harmonic structure, formant, and envelope. Positive suppression is also supported in the literature on frontal-medial theta oscillations associated with the automatic processing of stimuli to prevent unnecessary attention allocation [58]. Early suppression of theta has been reported in older adults for visual memory processing linked to inhibitory control [59]. One explanation is that age-related reduction in processing speed delay the selection of irrelevant information causing more attention allocation at the onset of stimuli to irrelevant distracting information, resulting in early suppression of theta activity. Research suggests a link between the N1 latency and P1 amplitude and the suppression of theta [59]. Indeed, we previously showed significant differences in N1 and P1 amplitude after music and visual art training in the same set of listeners [43].

### 4.1. Limitations

This study had some limitations. First, there was a relatively small number of participants allocated to each group. Future studies should consider a larger sample size that reflects the diversity of the population. In addition, in the present study our groups were comprised of a large percentage of female participants. Therefore, it is important to balance biological sex and gender in subsequent studies. Finally, our study included short-term (three-month) arts-based interventions. Long-term music and visual art interventions may yield stronger and longer-lasting effects on cognition and quality of life.

### 4.2. Future Research

As mentioned above, future studies should consider recruiting a larger, more diverse sample of older adults to examine the effects of arts-based interventions at different time points. Researchers may also consider examining neural responses to arts-based interventions stratified by age to determine if responses differ throughout the lifespan. In addition, the inclusion of standardized cognitive measures and time-frequency responses will enable researchers to clarify the role of modulated neural activity in arts-based learning.

## 5. Conclusions

Our data extend prior work by further suggesting neural oscillation phase synchronization can index learning associated with music and visual art training. Older adults who completed both training regimens demonstrated enhancements in theta power as compared to controls for pitch and speech stimuli. These data provide important new evidence of neuroplasticity and mechanisms associated with learning music or visual arts in aging adults. Changes in theta and beta rhythms increased post-training as compared to controls and were sustained three months post-training.

While further studies are needed to elucidate the specific relationship between neural oscillations and different perceptual-cognitive mechanisms of learning, our findings suggest phase modulations may offer a novel approach to understanding how neural networks respond to interventions for rehabilitation of older adults.

## Figures and Tables

**Table 1:** Group Demographic Data.

	Music (*n* = 16)	Visual Art (*n* = 17)	Control (*n* = 17)
Age	67.75 (6.02)	68.47 (6.21)	68.53 (5.99)
Sex (M/F)	3/13	2/15	3/14
Education	16.38 (2.68)	17.18 (2.40)	16. 94 (1.48)

**Table 2:** Results from the clustered-statistic comparing auditory evoked responses elicited by deviant and standard stimuli from the music and speech oddball paradigm before the intervention.

Paradigms	Cluster	Range;	Peak	Electrodes	*p* Values
Music	1	90–265 ms	145 ms, FC3	Fp1, AF7, AF3, F1, F3, F5, F7, FT7, FC5, FC3, FC1, C1, T7, C5, C3, P5, P7, Fpz, AF8, AF4, AFz, Fz, F2, F4, F6, F8, FC6, FC4, FC2, FCz, Cz, C2, C4, C6, CP4	<0.001
	2	227–396 ms	371 ms, C6	F1, F3, FC3, FC1, C1, CP1, AF4, AFz, Fz, F2, F4, F6, F8, FT8, FC6, FC4, FC2, FCz, Cz, C2, C4, C6, T8, CP6, CP4, CP2	<0.001
	3	57–230 ms	156 ms, O1	CP5, CP3, CP1, P1, P3, P7, PO7, PO3, O1, Oz, POz, CPz, Cz, C2, C6, T8, TP8, CP6, CP4, CP2, P2, P4, P6, P8, PO8, PO4, O2	<0.001
	4	264–389 ms	375 ms, Oz	P5, P7, PO7, PO3, O1, Oz, POz, PO4, O2	<0.001
Speech	1	47–332 ms	213 ms, FC3	Fp1, AF3, F1, F3, F5, FC5, FC3, FC1, C1, TP7, T7, C5, C3, CP5, CP3, CP1, P1, P3, P5, P7, PO7, PO3, O1, Oz, POz, Pz, CPz, Fpz, Fp2, AF8, AF4, AFz, Fz, F2, F4, F6, FC6, FC4, FC2, FCz, Cz, C2, C4, C6, T8, TP8, CP6, CP4, CP2, P2, P4, P6, P8, PO8, PO4, O2	<0.001
	2	225–400 ms	359, TP7	Fp1, AF7, AF3, F1, F5, F7, FT7, FC5, FC3, FC1, C1, TP7, T7, C5, C3, CP5, CP3, CP1, CPz, Fpz, Fp2, AF8, AF4, AFz, Fz, F2, F4, F6, F8, FT8, FC6, FC4, FC2, FCz, Cz, C2, C4, C6, CP2	<0.001
	3	61–244 ms	219, PO8	P7, PO7, O1, Oz, F8, FT8, C6, T8, TP8, CP6, P6, P8, PO8, PO4, O2	=0.002

**Table 3:** Results from the clustered-statistic comparing oscillatory activity elicited by deviant and standard stimuli from the music and speech oddball paradigm before the intervention.

Paradigms	Frequency Band	Cluster Difference	Range	Peak	Electrodes	*p* Values
Music	Theta	1; D > S	125–275 ms	175 ms, T8	FT8, T8, TP8	=0.009
		2; D < S	150–200 ms	175 ms, FCz	FC1, FC2, FCz	=0.032
	Alpha					
	Beta	1 D < S	150–275 ms	225 ms, AFz	AF3, AF4, AFz, Fz, F2	=0.014
	Gamma					
Speech	Theta	1; D > S	75–275 ms	200 ms, POz	Fp1, AF7, AF3, F1, F3, F5, FC5, FC3, P1, P3, PO3, O1, Oz, POz, Fpz, Fp2, AF8, AF4, AFz, Fz, F2, F4, F6, F8, FT8, FC6, C6, T8, TP8, CP6, P2, P4, P6, P8, PO8, PO4, O2	<0.001
		2; D < S	175–225 ms	200 ms, FC1	FC3, FC1, FC2, FCz, C2, C4	=0.039
	Alpha	1; D > S	75–225 ms	200 ms, POz	Fp1, AF7, AF3, F1, F3, F5, FC5, FC3, FC1, C1, TP7, T7, C5, C3, CP5, CP3, CP1, P1, P3, P5, P7, PO7, PO3, O1, Oz, POz, Fpz, Fp2, AF8, AF4, AFz, Fz, F2, F4, F6, F8, FT8, FC6, FC4, FC2, FCz, Cz, C2, C4, C6, T8, TP8, CP6, CP4, P4, P6, P8, PO8,PO4, O2	<0.001
	Beta					
	Gamma	1; D < S	75–225 ms	150 ms, P7	C3, CP3, CP1, P1, P3, P5, P7, POz, Pz	=0.035

## Data Availability

Data can be found at https://osf.io/kuheq/ (accessed on 4 August 2022).

## Abbreviations

The following abbreviations are used in this manuscript:

ERP	Event-related potentials
EEG	Electroencephalography
MMN	Mismatched Negativity
TSE	Temporal-Spectral Evolution
